# Delayed Correlations Between Geomagnetic Activity and Human EEG Alpha and Theta Oscillations: Evidence from Archival and Experimental Data

**DOI:** 10.3390/brainsci16060547

**Published:** 2026-05-22

**Authors:** Carlee D. Chezzi, Kevin S. Saroka, Kate S. Branigan, Samuel J. Levac, Blake T. Dotta

**Affiliations:** Behavioural Neuroscience & Biology Programs, School of Natural Science, Laurentian University, Sudbury, ON P3E 2C6, Canada

**Keywords:** electroencephalography (EEG), geomagnetic activity, Kp index, alpha oscillations, theta oscillations, magnetoreception, electromagnetic field exposure, delayed neural effects

## Abstract

**Highlights:**

**What are the main findings?**

- Theta and low-alpha EEG activity showed significant delayed associations with geomagnetic activity, with the strongest correlations occurring about 19 days before EEG measurement.
- Simulated geomagnetic storm exposure produced significant reductions in theta, low-alpha, and high-alpha power 19 days later in an independent sample.

**What is the implication of the main finding?**

- Geomagnetic variation may exert delayed effects on human brain oscillations, extending beyond immediate exposure windows.

**Abstract:**

Neural oscillations in the alpha and theta bands have been linked to environmental factors, including geomagnetic disturbances, yet the temporal dynamics of these interactions remain poorly understood. This study examined the relationship between geomagnetic activity, quantified by the Kp index, and brain activity in low alpha (7–10 Hz) and theta (4–7 Hz) bands using two complementary approaches. In Experiment 1, archival EEG data from 238 subjects collected over four years were analyzed for correlations between daily Kp values and band power across a ±90-day window. Significant positive correlations (*p* < 0.01) emerged in both bands, with a spatially coherent peak in caudal regions occurring 19 days prior to EEG measurement. In Experiment 2, an independent sample of 22 participants was exposed to a simulated geomagnetic storm, and EEG was recorded at baseline and 19 days post-exposure. Paired-samples *t*-tests revealed significant within-subject reductions in theta, low alpha, and high alpha power over frontal and parietal regions, consistent with a delayed neural response. Together, these findings provide converging correlational and experimental evidence for a lagged influence of geomagnetic activity on human brain oscillations. The 19-day delay observed in both datasets suggests that geomagnetic disturbances may exert residual effects on neural dynamics well beyond immediate exposure, warranting further investigation into underlying mechanisms and potential behavioral relevance.

## 1. Introduction

Geomagnetic activity, driven by solar events such as solar flares and coronal mass ejections (CMEs), is a key component of space weather. These events can substantially alter Earth’s geomagnetic environment [1], disrupting communication systems, satellites, and power grids, and producing auroras at unusually low latitudes [2,3,4]. The magnitude of geomagnetic storms is quantified using the Kp index, ranging from 0 to 9, with higher values indicating stronger disturbances [5]. As Solar Cycle 25 approaches its peak, solar activity is expected to intensify, increasing the frequency and severity of such events [6].

Beyond their technological impacts, geomagnetic disturbances have been linked to diverse physiological outcomes. Studies have reported associations with cardiovascular changes, including altered heart rate variability, blood pressure fluctuations, and increased incidence of myocardial infarction [7,8,9], as well as shifts in psychiatric and behavioral states, with some works noting increased anxiety, depression, and suicide risk during geomagnetic storms [10,11,12]. A growing body of evidence also implicates space weather in neural dynamics [13,14]. Electrophysiological studies have observed EEG changes during geomagnetic disturbances [15,16], including work replicating Azerbaijani findings of correlations between right hemispheric activity and geomagnetic variation, particularly in the gamma and theta bands [16].

EEG alterations have frequently been reported in the alpha (7–13 Hz) and theta (4–7 Hz) ranges during periods of geomagnetic disturbance. Recent experiments have demonstrated human sensitivity to controlled manipulations of the geomagnetic field, with Wang et al. (2019) showing reproducible alpha-band changes when the ambient field was rotated [17]. These studies suggest that neural oscillations can be modulated by geomagnetic factors, yet the temporal structure of such effects remains poorly characterized. Most prior work has examined immediate or short-term responses, leaving the possibility of delayed brain–geomagnetic relationships largely unexplored.

In the present study, we first analyzed an archival EEG dataset (238 recordings collected over four years) to test whether geomagnetic activity, indexed by Kp, exhibited time-lagged associations with low alpha and theta power. This exploratory analysis revealed a spatially coherent correlation pattern peaking at ~19 days. To evaluate whether this delay could be reproduced under controlled conditions, we conducted a laboratory experiment in which participants were exposed to a simulated geomagnetic storm, with follow-up EEG measurements 19 and 20 days later. This combined observational–experimental approach was designed to assess the robustness of the delayed relationship and to advance understanding of how environmental electromagnetic conditions may shape neural dynamics over extended timescales.

## 2. Materials and Methods

### 2.1. Experiment 1: Archival Analysis

For this experiment, alpha wave activity was broken down into two categories for deeper investigation: Alpha1 or Low Alpha Activity (7–10 Hz), and Alpha2 or High Alpha Activity (10–13 Hz). Alpha activity is the predominant band measured in an adult human brain, both at rest and when active. Theta activity was measured between 4 Hz and 7 Hz for this study. As outlined by the National Oceanic and Atmospheric Administration (NOAA), the official Kp index serves as a globally averaged measure of geomagnetic activity worldwide, calculated by taking a weighted average of K-indices from a specified network of geomagnetic observatories known as the official Kp network. These values range on a scale from 0 to 9, where a score of 0 represents very little geomagnetic activity and a score of 9 represents an abundance of strong geomagnetic activity. The maximum deviation of the horizontal fluctuation of the electromagnetic fields is measured through a magnetometer and 3 h averages are reported through NOAA.

#### 2.1.1. Experimental Design

All research presented has been approved by the Laurentian University Research Ethics Board (LUREB) under file number 6019407. All EEG materials being discussed were purchased from Bio-Medical. A total of 184 participants were included in this study, with some being measured multiple times, with all cases occurring between 1 June 2009 and 5 April 2013, resulting in a total *N* of 238. Each participant was seated in a Faraday cage and had an EEG cap applied following standard procedure. The EEG recordings occurred within the Faraday cage to avoid interference from external electromagnetic activity that could impact the accuracy of the readings. Once seated, the EEG cap was placed on the head of the participant where electroconductive gel was administered via blunt syringe to each of the 19-electrode sensors. EEGs in all cases were recorded using a 19-electrode electroencephalography (EEG) cap that was connected to a Mitsar 201 amplifier and processed through the WinEEG software 3.11.24. Each of the 19 sensors followed the 10–20 International Standard of Electrode Placement, where measurements of electrical activity were recorded from the Fp1, Fp2, F7, F3, Fz, F4, F8, T3, C3, Cz, C4, T4, T5, P3, Pz, P4, T6, O1, and O2 regions of the brain. From here, the study began with the participants closing their eyes for two minutes while their EEG was recorded as a pre-baseline measure of activity. Each participant was then exposed to a novel field intervention of varying nature, but it must be emphasized that all data for this study was obtained before these exposures. In each case, EEGs were recorded under standard procedure where electrical resistance (Z) was conserved under 5 kOhms, data was filtered between 1.5 and 50 Hz with a notch filter set to 35–55 Hz. It is also worth noting that the majority of cases were sampled at a rate of 250 Hz, but some were sampled at a rate of 500 Hz. Cases where electrical activity was sampled at 500 Hz were resampled to 250 Hz for consistency within the dataset.

From each of these 238 records, 16-s segments from each pre-baseline eyes closed measure were extracted. This raw time-series EEG data was then imported into the MATLAB software for further analysis. This primarily included spectral decomposition of the EEG activity from each of the 19 sensors into the 9 typical frequency bands of brain activity: delta (1.5–4 Hz), theta (4–7 Hz), low alpha (7–10 Hz), high alpha (10–13 Hz), low beta (13–20 Hz), medium beta (20–25 Hz), high beta (25–30 Hz), low gamma (30–35 Hz) and high gamma (35–40 Hz). This decomposition was completed using the bandpower.m script available within the EEGLab toolkit. From here, all data was imported to both Microsoft Excel and SPSS software.

#### 2.1.2. Data Collection

To simplify the analysis process, all 238 EEG cases were transposed into one Excel file where columns included participant number, date of recording, Kp daily average for a total of 90 days before the date of the individual EEG, and a column for each of the recordings of brain activity from all 19 channels within each of the nine frequency bins. Each row represented an individual EEG participant case. This resulted in 263 columns and 239 rows. Data for the Kp daily averages on all 90 individual days leading up to the specific date of all 238 EEGs was extracted from the previously curated and sequential Kp Daily averages file and transposed into the current dataset for each EEG case. All Kp data was originally obtained from the National Oceanic and Atmospheric Administration (NOAA) online K-indices database, and daily averages were extracted for curation purposes.

#### 2.1.3. Statistical Analysis

All data was analyzed using the SPSS 27 software and MATLAB R2024a. For each case, the daily Kp average for each of the individual 90 days leading up to the recorded EEG and each of the 90 days following were correlated with measured brain activity within each of the 9 bands and 19 sensors. This was completed in SPSS through Pearson correlation, where a significance of less than 0.05 was verified with Spearman’s Rho.

All data was also imported to MATLAB and used to create all presented figures. Creation of the correlograms required the coded correlation of each Kp value for the 180 total days with each brain activity measurement as described above. From this point, a matrix was created between each channel, where Spearman correlations with a specified significance of either *p* < 0.05 or *p* < 0.01 were reported and all others were coded as being 0 or null. These refined datasets for each band were then used to create correlograms where correlation strengths between −0.15 and 0.2 were plotted, based on the sensor, on the *y*-axis and the number of days before or after the recorded EEG served as the *x*-axis.

For visualization and pattern identification, only correlations meeting the *p* < 0.01 threshold were retained, and non-significant correlations were masked in the correlograms. The primary aim was to identify spatially and temporally consistent patterns across channels, rather than isolated significant values. To facilitate transparency and independent inspection, an interactive dataset was created containing correlograms and scatterplots for each EEG channel across the ±90-day lag/lead window (180 days total). This tool allows users to select any sensor and examine the full correlation structure and corresponding scatterplot.

### 2.2. Experiment 2: Laboratory Simulation

#### 2.2.1. Simulated Geomagnetic Storm

The electromagnetic field used in this experiment was developed to simulate the sudden onset of a geomagnetic storm and has been described in previous studies [18]. This field comprises 5071 points plotted by voltage relating to the Kp daily fluctuations over a given period. The field could be divided by durations where the first 14 peaks and troughs occur over 600 ms and comprise 200 points and the second set of 14 cycles occurred for 300 ms comprising 100 points. The final section of this field is the interface which consists of 500 points occurring over 1.5 s. Each of these points could be converted to an equivalent voltage between 0 and 256 by a digital-to-analog converter (DAC). From this DAC, the signal and frequency were transmitted directly to a pair of rectangular 112 cm × 125 cm Helmholtz coils separated by 65 cm. These racks were wrapped with 72 turns of 30-gauge wire, resulting in a total coil width of 13 cm. Participants were seated in a non-magnetic chair positioned centrally between the two coil assemblies. The chair position was kept consistent across participants, and the coils were aligned in the same slots for each exposure. The participant’s head was therefore positioned within the inter-coil exposure region. However, because participants differed in body size and seated posture, some individual variation in the exact anatomical position of the head and body within the exposure volume may have been present. The field generated by this configuration should therefore be understood as a coil-generated, time-varying magnetic exposure rather than a perfectly homogeneous field across the entire participant. With each participant, this protocol was run for 40 min, consisting of 156 cycles with a 1000 ms delay between cycles and a 1 ms delay between points. Each participant was exposed to the same time-varying geomagnetic-storm-like waveform, but the waveform was scaled to one of three maximum field amplitudes: 100 nT, 1.3 µT, or 4 µT. These values represent the peak intensities produced by the experimental coil setup, not constant field strengths maintained throughout the exposure. Because the Kp index is a dimensionless planetary index of geomagnetic disturbance, these amplitudes should not be interpreted as direct Kp-to-T conversions. Rather, the Kp-derived temporal structure was used to shape the waveform, while the applied amplitudes were selected to provide a graded range of low-intensity magnetic-field exposures. The laboratory-generated exposure was therefore intended to model selected temporal features of geomagnetic disturbance under controlled conditions, not to reproduce the full spatial and temporal complexity of a naturally occurring geomagnetic storm.

#### 2.2.2. Experimental Design

**Figure 1:**
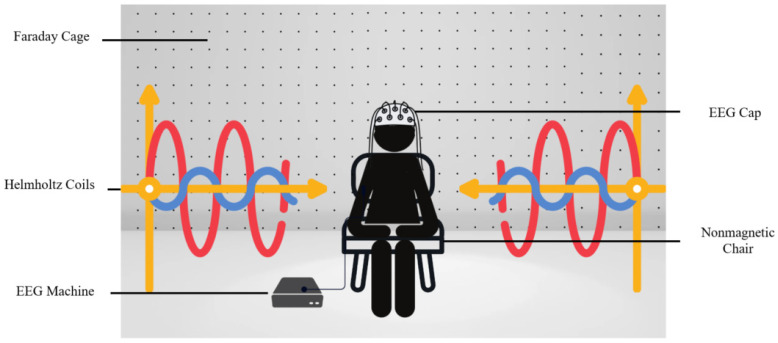
A visual illustration of the experimental setup from this experiment. The participant was seated in a non-magnetic chair to avoid influence on the production of the electromagnetic field. The non-magnetic chair was placed between two standing Helmholtz coils that were connected to a computer outside of the Faraday cage they were seated in. Through a digital-to-analog converter, the field was transmitted and applied through the coils, to the participant. The entire time, the participant was connected to the Mitsar by the EEG cap.

Each study participant was asked to come in for an EEG measurement three times. During their first visit, participants were provided with a consent form which debriefed them on the experimental protocol. Once consent was obtained, the participant was seated within the Faraday cage where the experiment would occur between the set of large roller coils mentioned previously. The participant was asked to remain seated for a duration of 45 min where a baseline of brain activity was recorded through electroencephalogram for 1 min with their eyes open followed by 1 min with their eyes closed. From here, the participant was notified the exposure period was starting and would last for a total of 40 min. Immediately following field exposure, the participant was once again asked to sit for 2 min where they had their eyes open for the first minute and their eyes closed for the second. The participant was then disconnected from the EEG setup and was scheduled to return on the 19th and 20th day following this initial exposure. On both days that each participant returned, they were seated in the same chair within the Faraday cage for a 10-min EEG recording. The participant was debriefed that no field exposure would occur during these sessions, and that they were being measured for baseline activity. This recording occurred under the same parameters as the prior recording, except the participant was asked to sit with their eyes open for five minutes, followed directly by 5 min of eyes closed recordings. A visual of the experimental design can be seen in Figure 1. The experimental dataset was also a between-subjects design, independent from Experiment 1. All participants were unique to this experiment and were measured only once per time point (Day 0, Day 19, Day 20).

#### 2.2.3. EEG Collection and Processing

All research presented has been approved by the University Research Ethics Board (xREB) under file number 6019407. All EEG materials being discussed were purchased from Bio-Medical. A total of 24 participants were included in this study, all being measured multiple times. All cases were recorded between 22 May 2024, and 27 June 2024. Each participant was seated in a Faraday cage and had an EEG cap applied following standard procedure. The EEG recordings occurred within the Faraday cage to avoid interference from external electromagnetic activity that could impact the accuracy of the readings. Once seated, the EEG cap was placed on the head of the participant where electroconductive gel was administered via blunt syringe to each of the 19-electrode sensors. All EEGs were recorded using a 19-electrode electroencephalography (EEG) cap connected to a Mitsar 201 amplifier and processed through the WinEEG software. Each of the 19 sensors followed the 10–20 International Standard of Electrode Placement, where measurements of electrical activity were recorded from the Fp1, Fp2, F7, F3, Fz, F4, F8, T3, C3, Cz, C4, T4, T5, P3, Pz, P4, T6, O1, and O2 regions of the brain. From here, the study began with the participants sitting with their eyes open for 1 min followed by sitting with their eyes for 1 min while their EEG was recorded as a pre-baseline measure of activity. This same baseline procedure was performed immediately following the simulated geomagnetic field exposure.

#### 2.2.4. Data Collection

The data were recorded at a sampling rate of 250 Hz and processed using WinEEG. Eye blink artifacts, identified as transient spikes (<1 Hz oscillations) over the Fp1 and Fp2 sensors, were corrected using independent component analysis (ICA). Following ICA, 10 s excerpts were taken from baseline measurements (both pre- and post-exposure) and exported as text files, which were then imported into sLORETA for spectral analysis. Using the Utilities application in LORETA, the text files were converted into sLORETA-compatible files. The electrode positions were transformed into coordinates via the “Electrode names to coordinates” function and then converted into a transformation matrix using the “Electrode coordinates to transformation matrix” function, which is based on the 3D profile of the human cerebrum. The EEG data text files were imported into the “EEGs to cross spectrum” function, where the number of electrodes, time frames per epoch (2500), and sampling rate were added. Computations were set to occur across all frequency bands (delta, theta, alpha1, alpha2, beta1, beta2, beta3, and gamma). The resulting cross-spectrum files and the computed transformation matrix were imported into “Cross spectra to sLORETA,” producing cross-spectral densities for analysis.

## 3. Results

### 3.1. Correlation Effects of Experiment 1

**Figure 2:**
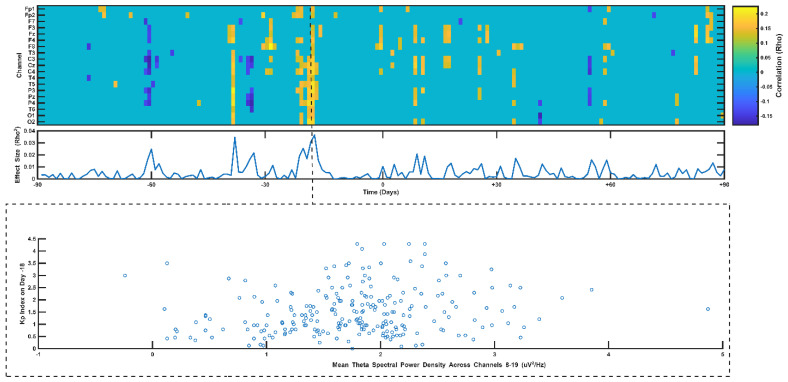
(**Top**) Correlogram depicting the significant theta correlations (*p* < 0.05) between spectral densities recorded from each channel with the Kp value measured between 90 days before and 90 days after the date when the EEG was obtained. (**Middle**) Time-series depicting the correlation (Spearman rho) between averaged caudal spectral densities within the theta band and each of the lag/lead Kp values. (**Bottom**). Scatterplot depicting the relationship between Kp index and the mean theta caudal spectral density on Day 19.

**Figure 3:**
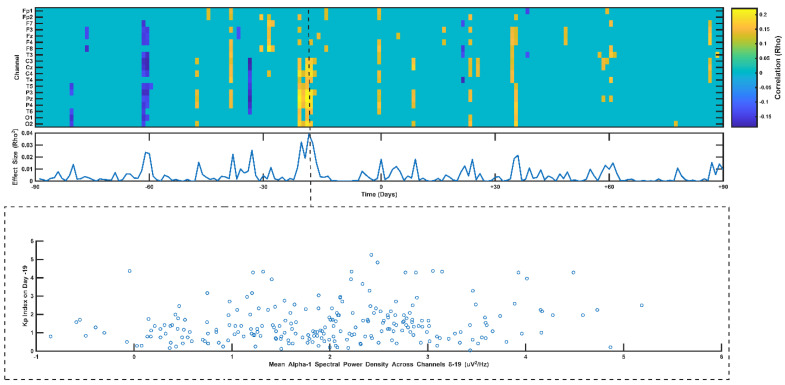
(**Top**) Correlogram depicting the significant alpha-1 correlations (*p* < 0.01) between spectral densities recorded from each channel with the Kp value measured between 90 days before and 90 days after the date when the EEG was obtained. (**Middle**) Time-series depicting the correlation (Spearman rho) between averaged caudal spectral densities within the alpha-1 band and each of the lag/lead Kp values. (**Bottom**). Scatterplot depicting the relationship between Kp index and the mean alpha-1 caudal spectral density on Day 19.

To examine the potential relationships between EEG activity and geomagnetic fluctuations, Spearman correlations were computed for each frequency band between spectral densities at each EEG channel and daily Kp values spanning 90 days before to 90 days after each recording. An a priori significance threshold was applied, whereby correlations were retained only if *p* < 0.01 and the effect size (ρ^2^) exceeded 0.03. When masked for non-significant results, distinct spatial and temporal patterns emerged in the theta and low alpha (alpha-1) bands, with pronounced clusters over posterior sites (Figure 2 and Figure 3, top panels). Interactive correlograms for individual channel–lag combinations are provided in Supplemental Figure S1.

**Figure 4:**
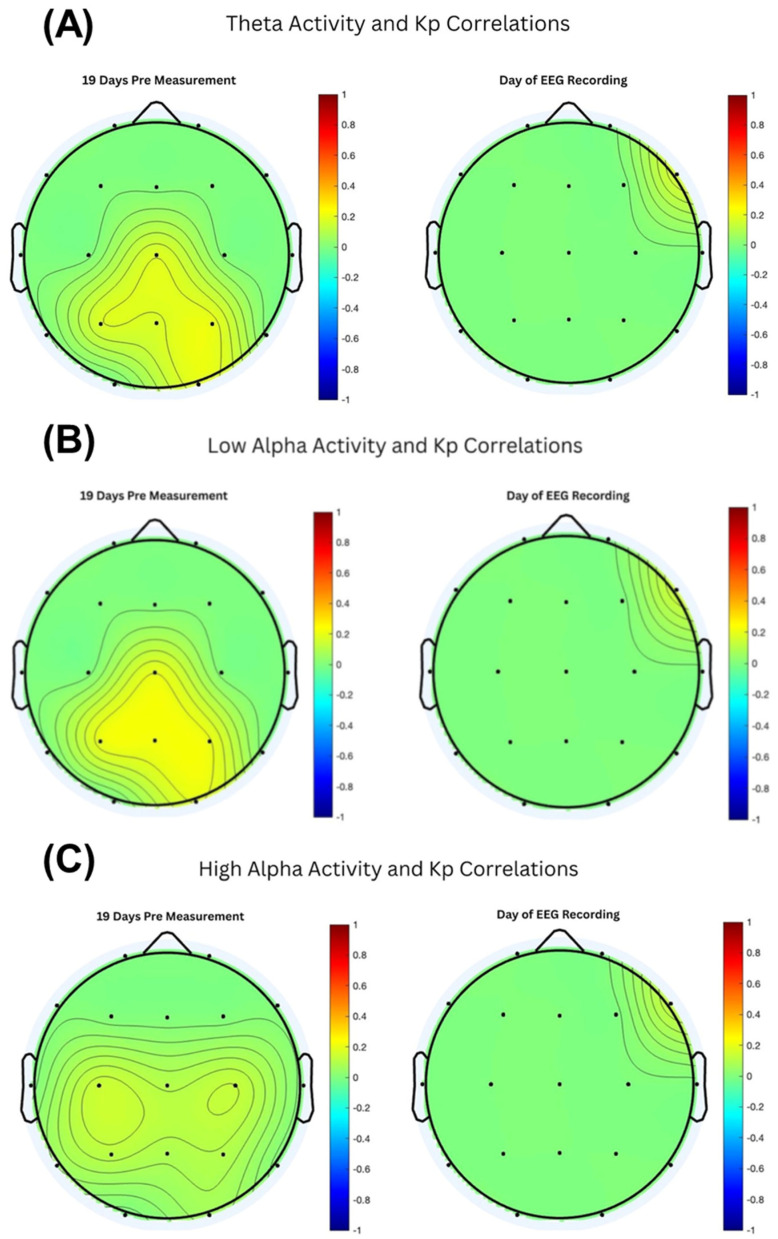
Topographic maps showing the spatial distribution of correlation coefficients derived from the relationship between daily Kp values and EEG band power at two time points: 19 days before EEG recording (left) and on the day of recording (right). Maps are shown for (**A**) theta (4–7 Hz), (**B**) low alpha (7–10 Hz), and (**C**) high alpha (10–13 Hz) bands. Warmer colors indicate positive correlations; cooler colors indicate negative correlations.

Given this clustering, mean spectral densities were computed for the caudal electrode set (T3, C3, Cz, C4, P4, T5, P3, Pz, P4, T6, O1, O2) and correlated with Kp at each lag/lead. Effect sizes plotted as a function of time relative to EEG acquisition (Figure 2 and Figure 3, middle panels) revealed a prominent increase peaking approximately 19 days prior to the recording date. The corresponding scatterplots (Figure 2 and Figure 3, bottom panels) illustrate these peak lagged relationships, which were strongest for the theta and low alpha bands. Topographic maps at this peak lag, compared to Day 0, are shown in Figure 4.

In this analysis, only correlations meeting the *p* < 0.01 threshold were considered, with the primary objective being to identify consistent spatial–temporal patterns rather than isolated effects. In the interest of full transparency, interactive datasets containing correlograms and scatterplots for all channels and lags/leads (±90 days) are provided in Supplemental Figures S1 and S2, allowing readers to inspect the complete correlation structure and explore effects across the entire recording window. While individual correlations were present across the full ±90-day range, these were not consistently localized to the same brain regions across lags. In contrast, the 19-day prior correlation pattern was both spatially coherent, most prominently in the caudal channels, and reproducible across frequency bands, which led us to select this time point for targeted experimental testing in Experiment 2.

### 3.2. Experimental Effects of Experiment 2

**Figure 5:**
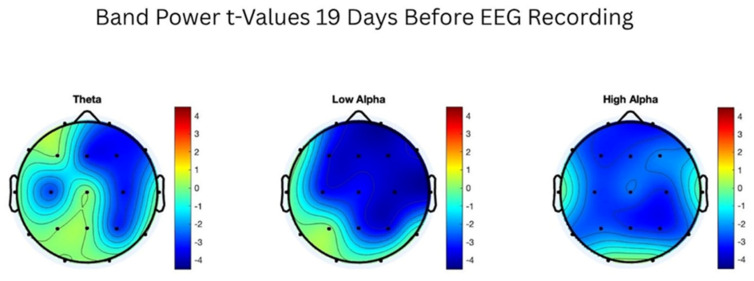
Topographic distribution of EEG band power *t*-values (Day 0 vs. Day 19) following exposure to a simulated geomagnetic field. Decreases in power were observed in the theta (4–7 Hz), low alpha (7–10 Hz), and high alpha (10–13 Hz) bands, particularly over right frontal and parietal regions. Negative *t*-values (blue) reflect reduced band power 19 days after exposure.

EEG data from 22 participants collected between 22 May and 27 June 2024 were analyzed to test for delayed effects of a simulated geomagnetic disturbance. Paired-sample *t*-tests compared band power (µV^2^/Hz) at Day 0 (baseline) and Day 19 post-exposure for theta (4–7 Hz), low alpha (7–10 Hz), and high alpha (10–13 Hz) bands. Topographic t-maps (Figure 5) revealed significant reductions in power across all three frequency ranges, with effects concentrated over right frontal and parietal regions. While smaller and less consistent changes were observed at Day 19, the strongest and most coherent effects occurred at the 19-day interval.

**Figure 6:**
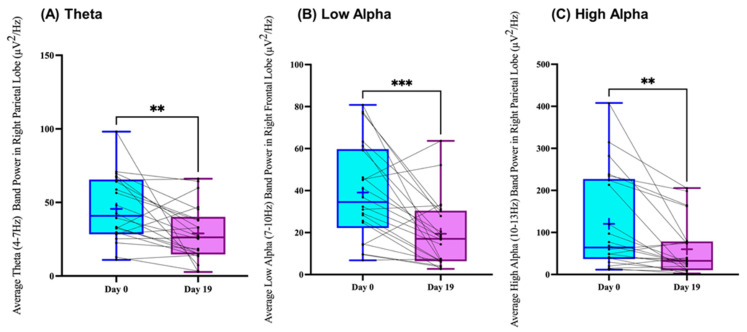
Within-subject changes in EEG band power 19 days following simulated geomagnetic field exposure. Boxplots show reductions in (**A**) theta (4–7 Hz) band power at the right parietal lobe, (**B**) low alpha (7–10 Hz) at the right frontal lobe, and (**C**) high alpha (10–13 Hz) at the right parietal lobe. Each line connects a single participant’s Day 0 and Day 19 values. All comparisons showed significant decreases (theta: *t*(21) = 3.03, *p* = 0.006; low alpha: *t*(21) = 3.93, *p* < 0.001; high alpha: *t*(21) = 3.25, *p* = 0.004). Error bars represent SEM. ** *p* < 0.01, *** *p* < 0.001.

To illustrate the magnitude and consistency of these changes within individuals, Figure 6 presents paired boxplots for representative electrodes in each band: theta at the right parietal lobe, low alpha at the right frontal lobe, and high alpha at the right parietal lobe. In each case, nearly all participants exhibited a decrease from baseline, and these within-subject drops were statistically significant (theta: *t*(21) = 3.03, *p* = 0.006, d = 0.65; low alpha: *t*(21) = 3.93, *p* < 0.001, d = 0.84; high alpha: *t*(21) = 3.25, *p* = 0.004, d = 0.69). These experimental results mirror the archival correlations from Experiment 1, providing convergent evidence for a delayed (~19 day) neural response to geomagnetic-like perturbations.

These findings indicate a consistent within-subject reduction in absolute EEG band power in the theta, low alpha, and high alpha ranges at approximately 19 days following simulated geomagnetic storm exposure. This pattern is in the opposite direction to the positive correlations observed in the archival dataset, where higher Kp values were associated with increased band power. Because the present experimental data reflect absolute power changes and did not include an intensity spectrum decomposition, the directionality difference with the archival correlations should be interpreted in the context of these methodological distinctions.

## 4. Discussion

Our study aimed to investigate the relationship between geomagnetic activity, quantified by the Kp index, and cerebral dynamics. The results revealed positive correlations between Kp values and both low alpha and theta band power, with a notable 19-day prior correlation in these bands across multiple brain regions. This pattern is consistent with the view that external geomagnetic factors can modulate neural activity, although the underlying drivers and their generalizability remain to be established.

Previous research supports these observations and highlights the larger context in which they occur. Mulligan et al. (2010) demonstrated that changes in right hemispheric EEG activity correlated with increases in geomagnetic activity, particularly in the gamma and theta bands [16]. Our findings are compatible with those reports, while also indicating that low alpha activity may be similarly affected. The observed 19-day lagged effect, particularly in the theta band, points toward a potentially complex temporal relationship between geomagnetic disturbances and neural activity. Comparable evidence comes from Wang et al. (2019), who demonstrated changes in alpha-band EEG activity under controlled alterations of the ambient geomagnetic field [17]. Such experimental demonstrations help to contextualize the correlations observed here. The positive associations between Kp values and EEG activity align with earlier research linking geomagnetic activity to physiological and psychological changes, such as alterations in heart rate variability, blood pressure, and certain health outcomes [19,20].

The prominence of the 19-day prior correlation in the theta band is notable given theta’s established role in large-scale network coordination and memory encoding [21]. Theta oscillations are particularly sensitive to neuromodulatory and environmental inputs, including stress, fatigue, and circadian factors [22,23], which could plausibly be influenced by geomagnetic conditions. A delayed association of this kind may indicate that geomagnetic disturbances set in motion slower-acting neurophysiological processes that only manifest in measurable oscillatory changes weeks later. The mechanism underlying the approximately 19-day delay remains unknown. The present data do not allow us to determine whether this delay reflects a direct neural effect of geomagnetic-like exposure or an indirect change in brain-state regulation unfolding over longer timescales. Several non-exclusive possibilities warrant consideration. First, geomagnetic disturbance may interact with sleep or circadian-regulatory systems, which could alter resting alpha and theta activity over subsequent days or weeks. Second, the effect may reflect slower network-level adaptation or homeostatic reorganization, rather than immediate modulation of EEG power, with measurable differences emerging only after repeated sleep–wake cycles. Third, the delay may reflect interaction with broader endogenous biological rhythms that were not measured in the present study. These possibilities should be treated as hypotheses rather than conclusions.

In the experimental arm, decreases in alpha and theta power were observed 19 days after simulated geomagnetic storm exposure, particularly in right frontal and parietal regions. Alpha oscillations are implicated in attentional and cognitive processes, while theta oscillations are often associated with memory and integrative network activity. The co-occurrence of reductions in both may indicate a coupling mechanism between these bands, consistent with prior work on cross-frequency interactions [24]. While the present design cannot determine causality, the concordance between archival and experimental data suggests that the delayed timing effect warrants further investigation.

The observed alpha and theta changes are also consistent with the literature on environmental electromagnetic influences on cortical excitability [25]. However, the absence of behavioral measures in this study means that we cannot determine whether these neural changes translate into measurable cognitive or affective outcomes. Future studies should integrate behavioral assessments alongside EEG to evaluate potential functional significance.

It is noteworthy that the experimental simulation data yielded significant effects in the opposite direction of the archival correlations. Specifically, while the archival analysis showed positive correlations between Kp and EEG band power, the simulated geomagnetic storm produced significant reductions in absolute band power. These differences may reflect distinctions between correlational field data and controlled exposure conditions, including the absence of an intensity-spectrum analysis in the present study, which limits direct comparison of spectral shape changes. It is also possible that the reductions observed in the experimental condition reflect transient suppression or network-level reorganization not captured by gross power correlations in the archival dataset. Further work using spectral density distributions and within-band spectral slope analyses would be required to resolve these apparent directional differences.

Given the complexity of brain–environment interactions, these results should be interpreted cautiously. Potential confounding factors, including other environmental variables, individual susceptibility, and compensatory neural mechanisms, could all influence the observed relationships. Larger and more controlled studies, incorporating varying exposure intensities and durations, will be needed to clarify the robustness and scope of these effects. Future work should extend these findings using longitudinal designs with repeated EEG sampling across the days and weeks following natural or simulated geomagnetic disturbances. Such studies should directly compare naturally occurring geomagnetic storms with laboratory-generated field patterns, while also incorporating behavioral, cognitive, and physiological measures to determine whether delayed changes in alpha and theta activity have functional consequences. Finally, future experiments should systematically vary field intensity, waveform structure, and exposure duration to determine which physical features of geomagnetic-like stimulation are most relevant to delayed EEG modulation.

## 5. Conclusions

This two-part study provides converging evidence from archival and experimental data that geomagnetic activity is associated with changes in human neural oscillations, particularly in the low alpha and theta bands. Our results extend earlier work by demonstrating a reproducible delayed relationship, peaking at approximately 19 days, between geomagnetic disturbances and EEG activity. While the present findings show clear neural associations, we did not assess behavioral or cognitive outcomes, and the functional significance of these changes remains undetermined. These results position geomagnetic variation as an environmental factor that is capable of influencing neural dynamics over extended timescales and highlight the need for further investigation into its mechanisms and relevance.

## Data Availability

The data that support the findings of this study are not publicly available due to the conditions of the ethics approval and participant confidentiality requirements, but are available from the corresponding author upon reasonable request and with appropriate institutional approval.

## Supplementary Materials

The following supporting information can be downloaded at: https://www.mdpi.com/article/10.3390/brainsci16060547/s1, Figure S1: Interactive channel-by-day correlation heatmap (theta band); Figure S2: Interactive channel-by-day correlation heatmap (low-alpha band).
