# Supplementary material for: Delayed Correlations Between Geomagnetic Activity and Human EEG Alpha and Theta Oscillations: Evidence from Archival and Experimental Data
Source: Brain Sci. 2026 May 22;16(6):547. doi: 10.3390/brainsci16060547 (PMC13296945; doi:10.3390/brainsci16060547)
Supplement: Supplementary file 1 [file brainsci-16-00547-s001.zip › brainsci-4248476-supplementary/Sup_Fig_Heatmap/Sup Figure S1 theta_interactive_heatmap_scatterplot.html]

Interactive Heatmap and Scatterplot


# Interactive Heatmap and Scatterplot
